# Behavioral and Cultural Insights, a Nationwide Study Based on Repetitive Surveys of WHO Behavioral Insights Tool in Greece Regarding COVID-19 Pandemic and Vaccine Acceptance

**DOI:** 10.3390/ijerph20010216

**Published:** 2022-12-23

**Authors:** Panagiotis Prezerakos, Katerina Dadouli, Eirini Agapidaki, Christina-Maria Kravvari, Ioanna Avakian, Athanasia-Marina Peristeri, Lemonia Anagnostopoulos, Varvara A. Mouchtouri, Konstantinos N. Fountoulakis, Sotirios Koupidis, Christos Hadjichristodoulou

**Affiliations:** 1 Department of Nursing, University of Peloponnese, 22100 Tripoli, Greece; 2 Laboratory of Hygiene and Epidemiology, Faculty of Medicine, University of Thessaly, 41222 Larissa, Greece; 3 Secretary General of Public Health, Ministry of Health, 10433 Athens, Greece; 4 Ministry for Climate Crisis and Civil Protection, 15123 Athens, Greece; 5 3rd Department of Psychiatry, Division of Neurosciences, School of Medicine, Aristotle University of Thessaloniki, 54124 Thessaloniki, Greece; 6 Occupational and Environmental Health Sector, Public Health Policy Department, School of Public Health, University of West Attica, 11521 Athens, Greece

**Keywords:** COVID-19, repeated surveys, behavioral insights, vaccination hesitancy, vaccination intention

## Abstract

Monitoring behavioral and cultural insights during the pandemic is a useful tool to identify factors related to COVID-19 vaccine acceptance and confront the pandemic’s vast impact. Data were collected using a questionnaire designed according to the “survey tool and guidance” provided by the World Health Organization (WHO). Surveys were conducted by a market research company for five consecutive months, with a sample of 1000 individuals recruited per survey. Vaccination acceptance increased from 55.2% to 67.2%, while the percentage of undecisive individuals decreased from 16.3% to 10.6%. The proportion of vaccine resistant participants remained relatively steady (25–30%). Knowledge about the pandemic and compliance with preventive measures was high (>90%). Factors associated with vaccination included: Increased age, male gender, influenza vaccination, following authorities’ recommendations, being informed by HCWs or formal information sources, care for others, concern about the country’s economic recession and health system overload. Pandemic fatigue was reflected across the surveys, indicated by a decrease in the intention to self-isolate and remain at home when ill. Despite the decrease of undecisive individuals, a firm core of vaccine resistant individuals may be responsible for the relatively lower vaccine coverage compared to northern EU countries. Study results could be useful for developing approaches tailored to a reluctant population.

## 1. Introduction

The coronavirus disease (COVID-19) pandemic constitutes a global public health issue [1]. The first COVID-19 case in Greece was recorded in February 2020. One month later, the Greek government announced a general population lockdown to prevent the widespread transmission of the novel coronavirus. Since then, preventive measures have been modified several times according to the epidemiological data [2]. Two years later, the COVID-19 pandemic remains an ongoing public health issue taking varying shapes and forms.

In the absence of effective treatment and until the national vaccination rollout implementation, behavioral changes including physical distancing, frequent hand washing and facemask use played a crucial role in the battle against the pandemic. Several studies also revealed that, in addition to the preventive measures, knowledge and attitudes played an important role in reducing SARS-CoV-2 transmission by improving compliance with preventive measures [3,4,5,6,7]. In addition, relevant studies have shown that vaccination intention as well as population knowledge, attitudes and practices towards the pandemic fluctuate over time and according to epidemiological data [5,8,9,10,11,12]. Assessing this information and examining relative trends in the general population could provide a useful tool for developing public health strategies for current and future epidemiological threats. Variables related to the financial and psychological burdens of the pandemic are equally important indicators of health and society’s tolerance to pandemic control measures. Monitoring these variables provides a tool to establish the best rationalization for pandemic preventive policies [13].

Another crucial point in ending the pandemic was the development of safe and effective vaccines. Vaccination acceptance is challenging mainly due to the notable increase in ‘vaccine hesitancy’. Vaccine hesitancy is defined as the delay in acceptance or the refusal of vaccines, despite availability of vaccination services [14,15]. Vaccine hesitancy is a universal issue and refers to any vaccination. The most recent experience with the H1N1 vaccine reveals that the general population raises concerns regarding any vaccine, especially a novel one [16,17]. While vaccination against COVID-19 has been available to the general population since 21 February, many people are still indecisive. Understanding the predictors and determinants influencing vaccination intention will play a key role in public health policy to optimize vaccination rates, particularly among priority social groups [12]. Notably, many studies consider that individuals’ intention to be vaccinated against COVID-19 develop longitudinal changes; several vaccination-related population groups may emerge sharing similar behavioral characteristics, requiring additional attention or a tailored approach [18,19].

Concerning Greece, most studies regarding knowledge, attitudes, practices and the intention to be vaccinated against COVID-19 have primarily focused on healthcare workers (HCW), as the first group prioritized for vaccination [20,21,22,23,24,25,26,27]. Aspects related to HCWs’ were considered of major significance. This group was expected to play an important role in vaccine consultation and dissemination during the first period of COVID-19 vaccinations among the general population, with HCWs serving as role models for society [26,27,28]. While consultations by HCWs motivated part of the population, other drivers continue to play various roles during the pandemic’s evolution and vaccination rollout. To address various behaviors among the general population related to the pandemic, Greek authorities implemented the COVID-19 Snapshot Monitoring (COSMO) project, in line with the recommendation of the World Health Organization (WHO) Regional Office for Europe. The project encourages the use of an appropriately developed Behavior and Cultural Insights (BCI) tool, to collect information about the pandemic through repetitive surveys [29]. The project aims to provide rapid, flexible and regular data regarding risk perception, behavioral insights and vaccination intention. The theory underlying the term BCI is that health behaviors could be treated like medical issues requiring diagnosis and treatment. Understanding barriers and facilitators may make it easier to design, adapt and implement policies promoting a healthier lifestyle to certain populations [29]. Considering the continuously changing epidemiological situation and limited previous experience with pandemics of this magnitude, monitoring insights and adapting policies when necessary seemed to be the only measure for evaluating and controlling the disease impact. Greece is one of the countries that implemented this project, by collecting data on a monthly basis for several consecutive months. Our study presents data from five surveys conducted under the framework of the COSMO project between January to June 2021. The objectives of our study in accordance with the Greek adjustment of the COSMO project were to: (1) Monitor variables crucial for population behavior during the pandemic and document longitudinal changes, (2) estimate the vaccination hesitancy and intention among the general Greek population, and (3) identify determinants and predictors of vaccination intention. Overall, study results were used to assess vaccination campaigns during the pandemic, increase vaccination coverage of the population, and evaluate the effectiveness of pandemic response measures.

## 2. Materials and Methods

### 2.1. Study Protocol

A nationwide panel study was conducted through five cross-sectional surveys, to determine the current state of behavioral insights in Greece related to the COVID-19 pandemic. The surveys were carried out by a market research company (MRB Hellas SA) on a monthly basis for five consecutive months. A sample of 1000 individuals were recruited per survey.

A geographically stratified sampling plan based on 13 regions (NUTS level 2) was applied to produce a representative sample, considering each region’s age and sex distribution. However, the actual number of collected samples differed from the pre-determined number of samples. Therefore, post stratification weights were applied based on each region’s population, in addition to sex and age distribution within each region according to the most recent census conducted in 2011. Since the sample was population representative, the applied post weighted factors were close to unity with almost no fluctuation.

Data were collected using online questionnaires (75% of sample) and telephone interviews (25% of sample). The sample was selected by sending an invitation e-mail to answer an online questionnaire to members of the Cint panel [30] aged 18 years or older. Telephone numbers were collected combining the 5-digit prefixes assigned by E.E.T.T. (Hellenic Telecommunications and Post Commission), [31] as well as the MRB Hellas telephone number processing software. This software delivers randomly selected numbers and is entitled “Call Center Management Software—Voxco”. The telephone number processing software is supplied with all 5-digit eligible prefixes issued by E.E.T.T. It generates telephone numbers from all possible combinations that could be generated from the five-digit prefixes, where random samples (telephone numbers) are being selected. The aforementioned technique ensures that the sampling frame coverage is at 100%. Individuals who did not respond to the invitation were replaced by others in the same stratum. Telephone interviews were deemed necessary, since it was considered that older individuals may be unfamiliar with the usage of online questionnaires [32].

### 2.2. Development of Surveys

From 7 November 2020, Greece enforced new measures and restrictions on movement and business activity. On 14 November 2020, primary schools and kindergartens were closed and replaced by distance learning [2]. Commercial shops and other facilities were allowed to re-open on 14 December 2020, while schools and restaurants remained closed [2]. By the end of 2020, 138,850 cases and 4838 deaths were recorded in the country [33].

**Figure 1:**
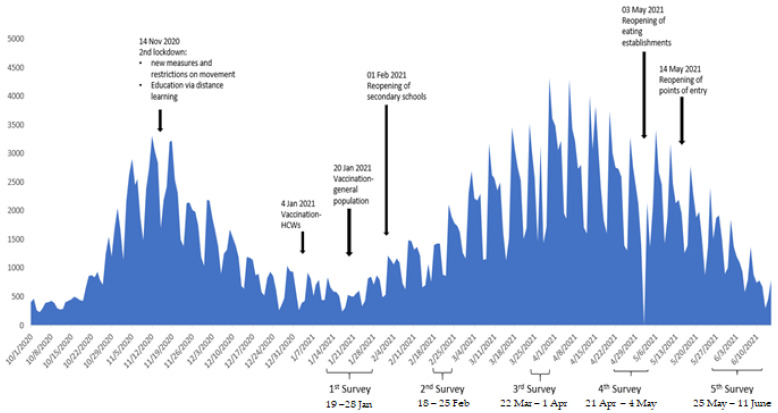
Sampling periods.

In the first survey (N= 1005), data were collected between 19–28 January 2021, following the country’s second COVID-19 pandemic wave (Figure 1). While measures and restrictions on movement and business activity were still in force, primary schools reopened on 11 January. Vaccination of HCWs began on 4 January [2]. By 19 January a total of 148,925 COVID-19 cases were reported and the number of deaths increased to 5488 [33]. The average daily incidence of the previous 14 days (15–28 January) was 51.7 per 1,000,000.

The second survey (N = 1008) was conducted between 18–25 February 2021, at the beginning of the third COVID-19 pandemic wave (Figure 1). Secondary schools reopened on 1 February and the vaccination of people aged 80 or older began towards the end of February [2]. Cases recorded by 18 February reached 176,059 with 6221 deaths [33]. The average daily incidence of the previous 14 days (12–25 February) was 121.2 per 1,000,000.

The third survey (N = 1000) was carried out between 22 March–2 April 2021, before the peak of the “third wave” in Greece (Figure 1). A total of 326,395 cases and 9788 deaths were recorded until 22 March [33]. The average daily incidence of the previous 14 days (19 March–1 April) was 253.8 per 1,000,000. During that period, mobility restrictions and capacity limitations in commercial establishments were maintained [2]. Vaccination rollout included individuals aged 60 or older, with limited access to Moderna and Pfizer vaccines [2].

The fourth survey (N = 1000) was conducted between 21 April–5 May 2021, during de-escalation of the third COVID-19 pandemic wave (Figure 1). On 4 March new measures were implemented, with the highest level placed on the entire country. In approximately half of the Greek prefectures, full closure of all schools and retail outlets was implemented [2]. The number of cases recorded on 21 April reached 323,639, with 9713 deaths [33]. The average daily incidence of the previous 15 days (21 April–5 May) was 201.8 per 1,000,000. COVID-19 vaccines were available to all adults over 30 years of age [2].

The fifth survey (N = 1000) was conducted between 25 May-11 June 2021 during the end of the third COVID-19 pandemic wave, when vaccination was available to all adults (29 May) [2]. The number of cases detected by 25 May reached 393,583 with 11,872 deaths [33]. The average daily incidence (25 May–11 June) was 122.0 per 1,000,000.

### 2.3. Variables

An online questionnaire was prepared to collect information on basic socio-demographic data, COVID-19 infection status, self-assessed health, prevention and behaviors, risk perceptions, information sources, restrictions, and vaccination intention [34].

### 2.4. Questionnaire

The questionnaire was designed based on the “survey tool and guidance” [34], which provides guidance to WHO European Region Member States wishing to conduct behavioral insight studies related to COVID-19. Validation and cross-cultural adaptation of the survey tool to the Greek language was conducted by experts from the field. The expert team was comprised of an epidemiologist, public health specialists, sociologists, an occupational health professional and a psychiatrist. All of them were native Greek speakers and highly proficient in English. One was a native speaker of English and highly proficient in Greek. Language validation was conducted by forward and back translation.

### 2.5. Data Analysis

Descriptive statistics were applied to all variables. For binary and categorical response options, the percentage of participants that selected each option was calculated. Mean and standard deviations were calculated for continuous variables. Data by surveys were compared using chi-square and Mann-Whitney tests. The association of vaccine intention with each variable was calculated using the Mantel-Haenszel test, which provides an estimate of the odds ratio of the independent/exposure variable, adjusted for the surveys (surveys 1–4). The association of vaccination intention with each variable was calculated using chi-square test (survey 5). Finally, we conducted multivariable analysis in the form of bivariate logistic regression analysis, as it allows assessment of the independent effects of multiple exposures on the vaccination intention, while controlling for confounding factors. Selection of variables for the bivariate logistic regression model was based on factors previously reported in the literature and found to be significant in the univariate analysis. All tests were two-sided and a *p* value of <0.05 was considered to indicate statistical significance.

There were two possible response options for study participants. Possible answers for the first response option included “yes”, “no” or “not applicable”. In the analyses, neutral responses were excluded (“not applicable”). For the second response option, participants rated their degree of agreement from one to seven. Response options were analyzed using two different methods. Using method 1, responses of “1” or “2” were considered as disagreement, responses “3”, “4” or “5” were considered as “neither agree nor disagree”, while responses “6” or “7” were taken as agreement. Using method 2, exploratory factor analysis was conducted to identify the optimal number of factors that could explain the variance. The number of factors retained was based on the scree plot and Kaiser’s criterion, i.e., eigenvalues being higher than one. If scree plot and eigenvalues led to different conclusions, the researchers decided upon the number of factors. Factor loadings above 0.30 were considered for the interpretation of the factors. Regression weights were saved for each retained factor and used in statistical analysis.

## 3. Results

### 3.1. Trends in Vaccination Intention

**Figure 2:**
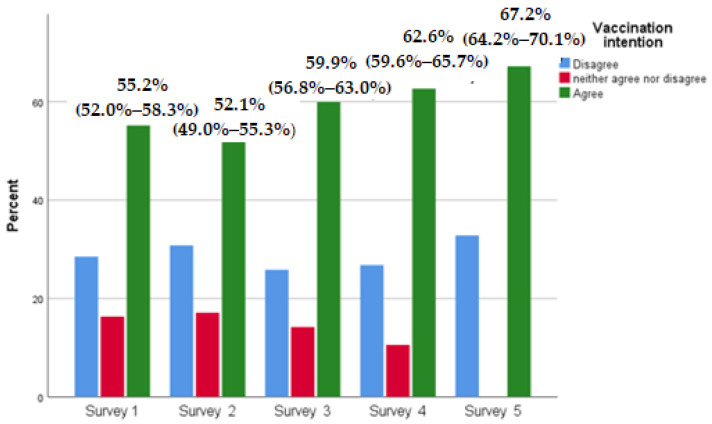
Responses concerning COVID-19 vaccination intention in five surveys implemented among the Greek adult population. The category “Vaccination intention: agree” includes participants who answered “5”, “6” or “7” to the question “If a COVID-19 vaccine becomes available and is recommended for me, I would get it”. The category “Vaccination intention: disagree” includes participants who answered “1”, “2” or “3”, while the category “Vaccination intention: neither agree nor disagree” includes participants who answered “4” to the same question “. In the fifth survey, the category “Vaccination intention: agree” includes those already vaccinated and non-vaccinated who answered “5”, “6” or “7” to the question “If a COVID-19 vaccine becomes available and is recommended for me, I would get it”. The category “Vaccination intention: disagree” includes participants who did not get vaccinated.

Trends in vaccination intention are depicted in Figure 2. Over five surveys there was an increase in the proportion of those answering “5”, “6” or “7” to the question “If a COVID-19 vaccine becomes available and is recommended for me, I would get it” (including those already vaccinated in survey 5), from 55.2% in the first survey to 67.2% in the fifth survey. In the fifth survey, the category “Vaccination intention: agree” includes those already vaccinated and non-vaccinated who answered “5”, “6” or “7” to the question “If a COVID-19 vaccine becomes available and is recommended for me, I would get it”. The category “Vaccination intention: disagree” includes participants who did not get vaccinated.

### 3.2. Socio-Demographic Characteristics

**Figure 3:**
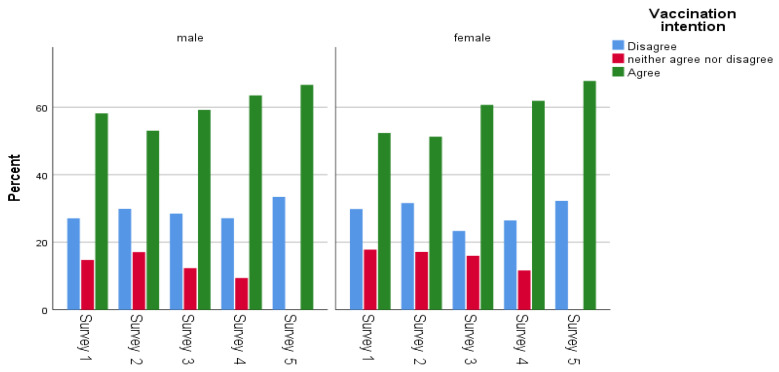
Gender and vaccination intention across the surveys.

**Figure 4:**
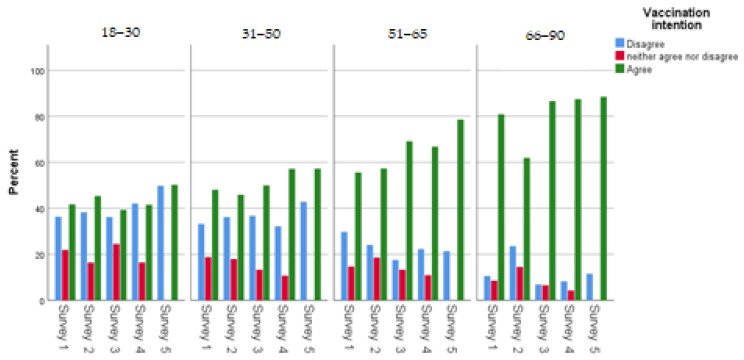
Age groups and vaccination intention across the surveys.

**Figure 5:**
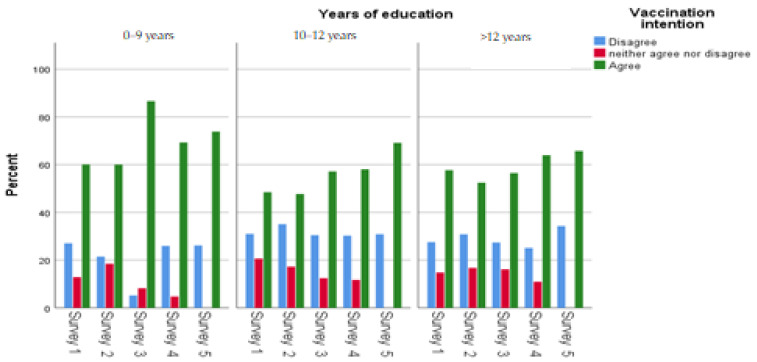
Years of education and vaccination intention across the surveys.

**Figure 6:**
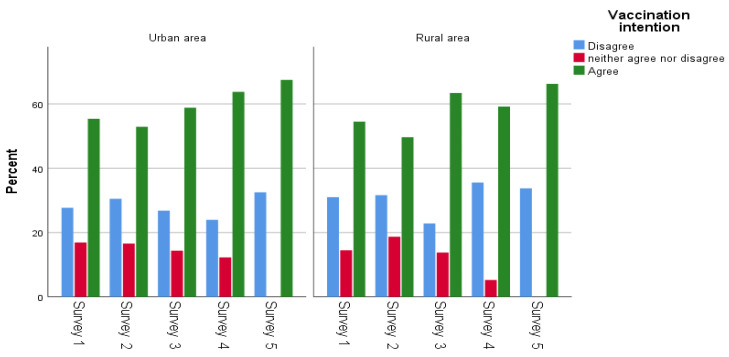
Area of residence and vaccination intention across the surveys.

Table 1 displays participants’ main socio-demographic characteristics. Women slightly outnumbered men in each survey. The mean age in the first survey was 47.5 (SD: 17.0; range: 18–90) years, 47.6 (SD: 16.5; range: 18–84) in the second survey, 48.0 (SD: 17.4; range: 18–89) in the third survey, 47.8 (SD: 17.4; range: 18–90) in the fourth survey and 47.9 (SD: 17.0; range: 18–91) in the fifth survey. Most sample participants had more than 12 years of education. The percentage of health professionals in each survey was approximately 15%, and the majority of participants lived in an urban area (75.6 in each round). Increased age (31–50: OR = 1.42, 51–65: OR = 2.54, 66–90: OR = 7.83), years of education (10–12 years: OR = 0.45, more than 12: OR = 0.60), and urban area (OR = 1.25) had a statistically significant association with COVID-19 vaccination intention (Table 1). However, regarding vaccination intention in the fifth survey, a statistically significant association was observed only between vaccination intention and increased age (31–50: OR = 1.50, 51–65: OR = 4.58, 66–90: OR = 10.28). Concerning vaccination intention and demographic characteristics per survey, vaccination intention increased steadily in most demographic subgroups; the highest proportion of vaccination intention were people aged 66 years and older (Figure 3, Figure 4, Figure 5 and Figure 6). Regarding the associations between socio-demographic characteristics and neutral position related to COVID-19 vaccination (compared to disagreement), the majority of associations were not statistically significant (Table S1—Supplementary Materials).

### 3.3. COVID-19 Infection Status

The percentage of confirmed infected participants, confirmed infected contacts of participants, and COVID-19 vaccination intention increased significantly across surveys (*p* = 0.006, <0.001 and <0.001, respectively). A statistically significant positive association was observed between the number of confirmed infected contacts of participants and COVID-19 vaccination intention (OR = 1.29 (1.09–1.53), *p* = 0.003). A negative association was observed between vaccination or intention to be vaccinated and infected participants in the fifth survey (Table S2—Supplementary Materials).

### 3.4. Prevention, Knowledge and Behaviours

According to Table 2, variation in the percentage of compliance with formal authorities’ recommendations was observed across the surveys (*p* = 0.001). However, approximately 30% of participants did not fully comply with instructions. There was also variation across the five surveys regarding the percentage of vaccination for influenza, self-isolation and disinfection of surfaces (*p* < 0.001, *p* < 0.001 and *p* < 0.001, respectively). In each survey, approximately 30% of participants declared the they used herbal supplements; the percentage of participants who consumed garlic, ginger and lemon to prevent or treat COVID-19 decreased across the surveys (*p* = 0.008).

Regarding vaccination intention in surveys one–four, a statistically significant positive association was identified between COVID-19 vaccination intention and good knowledge about symptoms (OR = 2.24), following the state’s recommendations (OR = 18.52), frequently washing hands (OR = 3.01), avoiding touching the eyes, nose and mouth (OR = 2.72), using disinfectants to clean hands (OR = 4.41), remaining at home when someone is sick (OR = 2.63), vaccinating for influenza (OR = 7.80), wearing a face mask (OR = 7.21), maintaining physical distance in public (OR = 5.02) and disinfection of surfaces (OR = 1.95). Additionally, it was observed that people who thought that they would become seriously ill due to COVID-19 were more likely to have the intention to get vaccinated (Table S3—Supplementary Materials). However, there was a statistically significant negative association between COVID-19 vaccination intention and use of herbal supplements (OR = 0.38), antibiotics (OR = 0.55), homeopathic treatment (OR = 0.51) and consumption of garlic, ginger and lemon (OR = 0.53). Concerning associations between preventive practices and a neutrality towards COVID-19 vaccination (compared to disagreement with vaccination), most associations have the same direction with lower measures of association (Table S4—Supplementary Materials). One exception is the use of antibiotics, which is more likely in participants who present a neutral position on vaccination (OR = 1.58 (1.22–2.04)) (Table S4—Supplementary Materials. Associations between vaccination intention (participants who have been vaccinated or intend to be vaccinated) in the fifth survey and preventive practices are similar and presented in Table 2.

According to Table 3, the percentage of respondents who informed how to care for a person belonging to a vulnerable group, searched information about competent authorities’ decisions on COVID-19 public health measures and the pandemic’s evolution in Greece, varied across the five surveys (*p* = 0.003, *p* = 0.005, *p* = 0.049, respectively).

Regarding vaccination intention in surveys 1–4, there was a statistically significant positive association between COVID-19 vaccination intention and searching information about the following: self and family protection (OR = 4.48), COVID-19 symptoms (OR = 4.28), scientific advancements in the development of COVID-19 vaccines or treatments (OR = 9.81), caring for a person who belongs to a vulnerable group (OR = 2.24), the difference between COVID-19 and influenza (OR = 3.21), the evolution of the pandemic situation (worldwide OR = 4.91, in Greece OR = 6.85), the authorities’ decisions (OR = 4.08), maintaining mental health during isolation (OR = 1.58) and preserving social contacts despite physical distancing (OR = 1.33). Concerning the association between knowledge about the pandemic and neutrality towards COVID-19 vaccination (compared to disagreement with vaccination), most associations have the same direction with lower measures of association (Table S5—Supplementary Materials). Associations between vaccination intention (participants who have been vaccinated or intend to be vaccinated) in the fifth survey and knowledge regarding the pandemic are similar and presented in Table 3.

Across the five surveys, the percentages of respondents varied relating to those who were informed mainly by state- and privately-owned television networks (*p* < 0.001 and *p* < 0.001), state and privately owned radio networks (*p* = 0.003 and *p* = 0.001), daily or weekly newspapers (printed) (*p* < 0.001), the Ministry of Health (*p* < 0.001), health care institutions (*p* = 0.017), opinion polls (*p* = 0.003) and VIPs (*p* = 0.001).

Regarding vaccination intention in surveys 1–4, the association between COVID-19 vaccination intention and information sources varied across age groups. People over 40 years of age were more greatly influenced by state owned television networks (≤40: OR = 5.53 (2.69–11.35), >40: OR = 20.29 (12.32–33.41)), by daily or weekly newspapers (printed) (≤40: OR = 4.75 (2.40–9.41), >40: OR = 16.90 (8.19–34.88)), by state owned radio networks (≤40: OR = 7.77 (3.36–17.98), >40: OR = 12.48 (7.53–20.66)), by the Ministry of Health (≤40: OR = 12.00 (7.24–19.88), >40: OR = 24.43 (16.50–36.17)), and by opinion polls (≤40: OR = 6.13 (1.37–27.41), >40: OR = 3.41 (1.69–6.89)). However, younger participants were influenced more by privately owned radio networks (≤40: OR = 8.73 (3.00–25.37), >40: OR = 4.47 (2.64–7.58)) and VIPs (≤40: OR = 6.13 (1.37–27.41), >40: OR = 3.41 (1.69–6.89)). Finally, older people appeared to be influenced positively by their family’s and friends’ opinion, while younger people appeared to be influenced negatively (≤40: OR = 0.51 (0.33–0.80), >40: OR = 1.43 (1.02–2.02)). (Table S6—Supplementary Materials).

Regarding vaccination intention in the fifth survey, the association between COVID-19 vaccination and information sources also varied across age groups. People over 40 years of age were influenced more by state owned television networks (≤40: OR = 0.78 (0.23–2.62), >40: OR = 13.65 (5.73–32.49)), by privately owned television networks (≤40: OR = 1.90 (0.56–6.43), >40: OR = 5.99 (2.49–14.38)), by daily or weekly newspapers (printed) (≤40: OR = 2.46 (0.66–9.09), >40: OR = 24.49 (3.32–180.80)), by state owned radio networks (≤40: OR = 3.56 (1.02–12.36), >40: OR = 10.28 (4.01–26.40)), by privately owned radio networks (≤40: -, >40: OR = 4.08 (1.55–10.75)), by opinion polls (≤40: OR = 1.64 (0.62–4.30), >40: OR = 5.61 (2.55–12.34)), by the Ministry of Health (≤40: OR = 3.20 (1.58–6.46), >40: OR = 21.55 (10.32–45.03)), by health care workers (≤40: OR = 4.78 (2.16–10.60), >40: OR = 12.53 (6.84–22.98)) and by health care institutions (≤40: OR = 4.20 (2.12–8.32), >40: OR = 23.45 (12.45–44.18)) (Table S6—Supplementary Materials).

### 3.5. Factor Analysis

Using data from surveys one–four, we conducted explanatory factor analysis using variables regarding information sources, with three factors identified. The first factor was labelled “mass media and social media”, the second factor “HCWs and states’ instructions” and the third factor “family’s, friends’ and colleagues’ opinion”. There was a positive statistically significant association between vaccine intention and individuals who trusted the information sources “mass and social media” and “HCWs and states’ instructions”. (Table S7—Supplementary Materials) Individuals 40 years of age or older appear to be more greatly influenced by mass and social media (≤0: OR = 1.49 (1.27–1.74); >40: OR = 1.62 (1.41–1.86)) and less influenced by HCWs’ and states’ instructions (≤40: OR = 6.06 (4.90–7.49); >40: OR = 5.69 (4.84–6.68)), compared to individuals up to 40 years of age. Finally, there was a negative statistically significant association between individuals who trust the opinions of their family members/friends/colleagues and vaccine intention in each age group (≤40: OR = 0.87 (0.74–1.02); >40: OR = 0.88 (0.78–1.00)) (Table S11—Supplementary Materials). We also conducted explanatory factor analysis using data which came from the fifth survey, with three factors identified. The first factor was labelled “HCWs, states’ instructions and public state mass media”, the second factor “private owned mass media and social media” and the third factor “family’s, friends’ and colleagues’ opinion”. There was a positive statistically significant association between individuals who trust the information sources “HCWs, states’ instructions and public state mass media” and vaccination (Table S9—Supplementary Materials). Individuals 40 years of age or older appear to be more greatly influenced by “HCWs, states’ instructions and public state mass media” (≤40: OR = 2.34 (1.75–3.12); >40: OR = 3.40 (2.71–4.28)) (Table S12—Supplementary Materials).

The main concerns of participants regarding the pandemic were similar across the four surveys: The wellbeing of loved ones, the saturation of the health care system and the economic recession (Table S12—Supplementary Materials).

We conducted explanatory factor analysis using data which came from surveys one–four, using variables related to the concerns. Four factors were identified: The first factor was labelled “My and my loved ones’ wellbeing and health system overload”, the second factor “Country’s economic recession”, the third factor “Personal financial problems” and the fourth factor “Losing my social life” (Table S9—Supplementary Materials). There was a statistically significant positive association between COVID-19 vaccination intention and the concern of “My and my loved ones’ wellbeing and health system overload” (OR = 2.83 (2.56–3.13). Moreover, there was a statistically significant positive association between COVID-19 vaccination intention and the “Country’s economic recession” (OR =1.19 (1.09–1.31)). However, there was a statistically significant negative association between COVID-19 vaccination intention, concern about “Personal financial problems” (OR =0.51 (0.49–0.56)) and “Losing my social life” (OR =0.71 (0.64–0.78)) (Table S14—Supplementary Materials).

We also conducted explanatory factor analysis using data that came from the fifth survey, using variables related to the concerns. Four factors were identified; the first factor was labelled as “My and my loved ones’ wellbeing”, the second “Country’s economic recession and health system overload”, the third factor “Personal financial problems” and the fourth factor “Losing my social life” (Table S10—Supplementary Materials). There was a statistically significant positive association between COVID-19 vaccination and the concern of “My and my loved ones’ wellbeing” (OR = 1.63 (1.42–1.88)) and the “Country’s economic recession and health system overload” (OR =1.51 (1.32–1.73)). However, there was a statistically significant negative association between COVID-19 vaccination and concern about “Personal financial problems” (OR =0.76 (0.66–0.87)) and “Losing my social life” (OR =0.58 (0.50–0.67)) (Table S15—Supplementary Materials).

### 3.6. Multivariate Analysis

According to multivariate analysis (Table 4) conducted using data coming from surveys one–four, there was a statistically significant positive association between COVID-19 vaccination intention and increased age (aOR= 1.010), male gender (aOR (male/female) = 2.08), 0–9 years of education (aOR (10–12 years/0–9 years) = 0.56), knowledge about COVID-19 symptoms (aOR = 1.63), acceptance of influenza vaccination (aOR = 4.97), following the recommendations of country authorities (aOR (Agree/Disagree) = 4.94); aOR (NAND/Disagree) = 2.88), being informed by mass media and social media (aOR = 1.45), HCWs’ and states’ instructions (aOR = 3.29), concern about their own and their loved ones’ wellbeing and health system overload (aOR = 1.60) and the country’s economic recession (aOR = 1.21). Conversely, there was a statistically significant negative association between COVID-19 vaccination intention and trusting information about the COVID-19 pandemic originating from family’s/friends’/colleagues’ opinion (aOR = 0.86), concern about personal financial problems (aOR = 0.80) and losing their own social life (aOR = 0.87). Regarding multivariate analysis among participants with a neutral position towards COVID-19 vaccination and vaccination deniers, many associations are statistically insignificant, while the remainder of the associations have the same direction (Table S16—Supplementary Materials).

According to multivariate analysis (Table 5) conducted using data from the fifth survey, there was a statistically significant positive association between COVID-19 vaccination intention (participants who have been vaccinated or intend to be vaccinated) and: increased age (aOR= 1.04), male gender (aOR (male/female) = 1.66), residence in large urban areas (>500,000 inhabitants) (aOR= 1.49), acceptance of influenza vaccination (aOR = 3.49), following the recommendations of the country’s authorities (aOR (Agree/Disagree) = 10.18; aOR (NAND/Disagree) =4.88), being informed by HCWs, states’ instructions and public state owned mass media (aOR = 2.23) and concern about country’s economic recession (aOR = 1.21). Conversely, there was a statistically significant negative association between COVID-19 vaccination and concern about personal financial problems (aOR = 0.77), as well as trusting information about the COVID-19 pandemic originating from family’s, friends’ and colleagues’ opinion (aOR = 0.83).

## 4. Discussion

The major findings emerging from our study across five repetitive surveys refer to changes in COVID-19 vaccination acceptance/hesitancy, trends in COVID-19 preventive practices and factors associated with COVID-19 vaccination acceptance among the general Greek population. The value of this study applies to examining population behavior during different stages of the pandemic, and identifying how factors related to COVID-19 vaccination developed under various circumstances. During the survey period, pandemic data and preventive social measures varied widely, from strict lockdowns to reopening of schools and commercial activity. The third round of the survey coincided with the peak of the third epidemic wave, which caused hundreds of deaths and lasted until late April 2021 [2]. Information from our study is of great importance for public health authorities. It can be used as an indicator for the effectiveness of implemented measures, as it documents changes in people’s attitudes and behaviors during the pandemic [13].

In line with the literature, we found that knowledge about COVID-19 symptoms in Greece was high and improved across the surveys. Moreover, compliance with preventive practices such as wearing face masks, washing hands, use of disinfectants and remaining home when ill remained high among the Greek population (more than 90% across surveys). Our findings are comparable with those from studies conducted in Europe, the USA and China [5,6,7,10]. However, our results revealed that over 30% of respondents faced difficulties strictly complying with national authorities’ recommendations. Moreover, during the research period, self-isolation and remaining at home while ill decreased, reflecting pandemic fatigue [35,36].

The highest percentage of compliance was observed in the first survey (January 2021). This finding may depict the challenges society faces with following national recommendations and the relative development of fatigue over time. Similarly, the proportion of participants who sought COVID-19 information daily declined over time, as well as the proportion of participants who addressed information means (television, social media, health workers, etc.). Respondents gradually lost interest in COVID-19. Another notable finding was the continued application of practices—such as herbal supplement use for COVID-19 prevention and consumption of garlic, ginger and lemon—by 30%–35% of the study population, even though their effect is controversial [37]. Regarding the psychological burden, our study revealed that the proportion of respondents with fears and concerns related to COVID-19 increased notably around the period of the third epidemic wave (third and fourth surveys).

During the study period, COVID-19 vaccine acceptance increased (from 55.2% in January /first survey to 67.2% in the 5th June survey). Meanwhile, the percentage of indecisive individuals decreased from 16.3% in first January survey to 10.6% in the 4th April survey. On the contrary, the percentage of participants who declined COVID-19 vaccination remained relatively steady at 25%–30% across all surveys. These results suggest that over time, indecisive individuals are more likely decide to get vaccinated, rather than vaccine refusers who maintain their decision. Our findings are in line with a similar Greek study, where the percentage of vaccine refusal was stable, despite the longitudinal increase of vaccine acceptance [38]. It is notable that the lowest percentage of COVID-19 vaccination refusal was recorded during the third survey, when 25.8% of respondents refused COVID-19 vaccination. The third survey coincided with the peak of the third pandemic wave in Greece; fear of rapid pandemic spread may have influenced the proportion of participants refusing vaccination. According to descriptive analysis, during the same period several psychosocial variables reached a higher rate. This indicates that COVID-19 vaccine hesitancy is not immutable, and that the pandemic impact could lead to behavior alterations. Surveys from the UK conducted during the first year of the pandemic agreed with the idea that vaccine receptibility fluctuated throughout the pandemic [18]. Similar findings were depicted in studies from Italy [8].

Our results suggest that vaccination intention in the general Greek population was lower compared to findings from studies conducted in Canada [39], the USA [40], Norway [41], France [42], South Africa [43], Portugal [44], Hong Kong [45], China [46], the UK [47] and Germany [48]. However, methods and timing of data collection varied widely from study to study. A survey in Greece following a similar sampling method conducted before the release of a COVID-19 vaccine (April and May 2020), depicted vaccination acceptance at 81.1% [4]. During the same period, a proportion of 57.7% of COVID-19 vaccine acceptant was mentioned in another Greek survey, where 16.3% of participants were indecisive. [49]. The results from a study based on four consecutive cross-sectional phone interview surveys (November 2020, February, April and May 2021) reported a vaccination intention rate that reached 84.6% in May 2021 [38]. However, in our study researchers utilized phone interviews and online questionnaires. This may have facilitated hesitant respondents to participate and more freely express negative opinions towards vaccination. Moreover, sampling in our study is more reliable, although convenient, as the samples of the surveys have been statistically equivalent to each other and stratified. The percentage of vaccination intention arising from our study was similar to the national vaccination rate, which was recorded one month later (first week of July 2021: 67.8%) when all vaccine types were available for the adult population [50].

According to multivariable analysis, results from surveys one–fourth revealed that age and sex are associated with COVID-19 vaccination intention; older and male participants are more willing to get a COVID-19 vaccine. More specifically, across the five survey rounds males were more vaccine acceptant than females, a finding which is in line with the majority of the literature [39,41,47,48,51,52,53]. However, it is notable that vaccination acceptance among women tends to increase over time. A closer look reveals that the highest proportion of indecisive females was reported during the third survey. A possible explanation for the sex association could address fertility issues raised during the period of the first surveys. These issues were resolved after the recommendation issued regarding pregnancy and COVID-19 vaccination [53,54].

Concerning the age factor, vaccination intention rate increased over time for individuals 18–65 years of age, while the proportion of indecisive participants declined. This finding is likely associated with the widespread use of COVID-19 vaccines globally, the number of studies supporting vaccine safety and efficacy, [55,56] as well as the implementation of restrictive regulations for unvaccinated individuals. Conversely, people 65 years of age and older were the most willing to be vaccinated across the five surveys. In accordance with the literature, age plays an important role in vaccination intention, especially in the European region [18,45,56,57].

Apart from age and sex, years and level of education appear to be associated with vaccination acceptance [38,42,47,58,59,60,61]. In our study, surveys one–four depicted a higher unwillingness among the population who completed 10–12 years of education, compared to respondents with 0–9 years and greater than 12 years of education. In contrast to previous work, our study supports that a higher education level is not associated with increased vaccination intention. This difference probably arises from the heterogeneity of educational backgrounds among respondents, since years of education does not always correspond to education level. Students of technological schools or universities study for more than 12 years in Greece, and may or may not attain postgraduate degrees such as a master’s or PhD. An analysis based on educational level, rather than years of education, may provide better insight to the role of education in vaccination acceptance by the Greek population.

According to most of the literature, vaccination intention is higher in urban areas. This is associated with a variety of factors including variations in views regarding the seriousness of COVID-19 infection, and intention to implement COVID-19 prevention strategies [62,63]. Our study also demonstrated association between residency area and vaccination intention. Indeed, vaccination rates are significantly higher in large urban areas, and that finding might be a consequence of effective compliance with public health measures. Increased compliance in metropolitan areas can be explained by better access to health care centers and possibly by the higher COVID-19 incidence resulting in critical overload of the healthcare system [64].

Concerning behavior and cultural characteristics related to vaccination acceptance (explanatory factor analysis), individuals who are altruists, care for others, comply with authorities’ recommendations and support collective responsibility are prone to COVID-19 vaccination. On the contrary, participants with self-serving behavior and more concerned about social and financials issues, were less acceptant or refused COVID-19 vaccination. In the literature, limited studies alluded to individuals’ profile regarding COVD-19 vaccine acceptance. Generally, results are in accordance with the findings of our study [18,48].

Regarding information sources, respondents trusting COVID-19 information from HCWs, formal authorities and public media are more likely to get the COVID-19 vaccine. Participants who rely on their family/friends’/colleagues’ opinion are less acceptant. Several studies depict similar findings [43,48,65,66]. Notably, individuals 40 years of age or older appear to be more greatly influenced by mass and social media. These individuals are less influenced by HCWs’ and states’ instructions compared to individuals up to 40 years of age.

According to the literature, vaccination for seasonal flu is a major predictor of COVID-19 vaccination [40,45,67,68]. In Greece, flu vaccination is recommended for older individuals (>60 years) and vulnerable groups. Thus, in our survey a question concerning seasonal flu vaccination was included in the “preventive practices “section, asking participants to state if they consider flu vaccination as a preventive measure towards COVID-19. As expected, positive answers in the aforementioned question were positively associated to COVID-19 vaccination intention in all surveys. During the 2020–2021 seasonal flu vaccination period, vaccine coverage for influenza among physicians in Greece was the highest observed in the last five years, according to the National Public Health Organization (EODY) [69]. This finding might reflect the identified attitude of our study related to seasonal influenza vaccination.

Concerning participants with neutral responses towards vaccination intention, analysis revealed that there were more receptive than hesitant respondents, although less receptive than the vaccinated participants on the majority of questions. This trend likely depicts the most easily influenced population regarding vaccine related initiatives, and thus the primary target group for vaccine promotion policies. Moreover, measures of association between knowledge/practices for COVID-19 prevention and neutral position on vaccination were estimated in the same direction, but lower than those with vaccination intention. This finding indicates a dose-response effect of the association between knowledge/practices for COVID-19 prevention with vaccination intention.

Survey findings proved particularly useful in policy-making decisions. Greek authorities and researchers were in constant communication in order to translate surveys results into pandemic control policies. As the surveys revealed, social media played an important role as an information source, especially among vaccine hesitant populations. To address this specific group, Greek authorities adapted their pandemic related campaign, which focused on television-based information—including social media communication—as their means of knowledge dissemination. Thus, short informative videos and vaccine related information were prepared and widely communicated through social media platforms, simultaneously confronting anti-vaccination allegations. During the last surveys, results revealed that participants became less interested in COVID-19 information. This finding may be related to mental fatigue caused by an overload of epidemiological data and social measure regulations. Based on this result, Greek stakeholders adjusted the frequency of COVID-19 informative television and radio broadcasts, protecting overexposure of experts and the population’s mental wellbeing. Moreover, daily pandemic briefings were scheduled to weekly briefings.

Our study has limitations. Firstly, selection bias could occur based on the method of questionnaire completion. This may lead to overestimation of positive attitudes towards the pandemic, as individuals with anti-vaccination views could refuse to participate. Moreover, individuals responding in a phone interview might express opinions which are agreeable to the interviewer and socially acceptable, but differ from their actual beliefs. However, the online questionnaire provided an option where respondents with negative attitudes towards the pandemic were protected by the privacy of an online procedure. Secondly, selection bias could concern population groups such as the Roma, who are less likely to participate in the survey although they may represent a notable percentage of the general Greek population; approximately 2% of the general population self-identify as Roma [70,71]. This population group is nomadic and continuously moving from one area to another, changing their personal contact information such as telephone number and address. Moreover, the vast majority lack computer literacy and are ineligible for an online survey. Finally, the last drawback of our study might be related to the survey questionnaire. Although it is an integrated tool concerning the pandemic, the questionnaire is quite long and could cause discomfort to participants. The order of questions could be revised, as significant questions related to COVID-19 vaccination intention are located in the middle or the end of the questionnaire, where participants are tired and less attentive.

## 5. Conclusions

COVID-19 vaccination intention in the general Greek population was at an intermediate level during the first months of 2021, following the initiation of the vaccine rollout. Although knowledge acquired about the pandemic and compliance with preventive measures during early 2021 was high, signs of fatigue appeared over time. Several factors are related to the decision of vaccination acceptance, and hesitant individuals could be influenced in their decision to get vaccinated. However, a vaccine resistant population group is difficult to address, as they reject communication through formal sources and act with more regard for private, rather than public interest. Further research could enlighten the dynamic of vaccine resistant groups and offer tools for a tailored approach.

## Figures and Tables

**Table 1:** Socio-demographic characteristics of the samples in each survey.

		Survey						Vaccination Intention		Vaccination or Intention to Be Vaccinated	
Variable	Categories	1	2	3	4	5	Comparison between Surveys	(Surveys 1–4)		(Survey 5)	
								OR 95%CI	Sig.	OR 95%CI	Sig.
Gender	Male	488 (48.5)	486 (48.5)	486 (48.6)	486 (48.6)	486 (48.6)	0.999	1.04 (0.88–1.22)	0.687	1.04 (0.81–1.35)	0.739
	Female	517 (51.5)	515 (51.5)	514 (51.4)	514 (51.4)	514 (51.4)		Ref.		Ref.	
Age groups	18–30	206 (20.5)	199 (19.9)	204 (20.4)	202 (20.2)	204 (20.4)	0.907	Ref.		Ref.	
	31–50	334 (33.2)	329 (32.9)	348 (34.8)	350 (35.0)	356 (35.6)		1.42 (1.12–1.80)	0.004	1.50 (1.06–2.13)	0.022
	51–65	270 (26.9)	267 (26.7)	245 (24.5)	238 (23.8)	237 (23.7)		2.54 (1.98–3.27)	<0.001	4.58 (3.05–6.88)	<0.001
	66–90	196 (19.5)	206 (20.5)	206 (20.5)	210 (21.0)	203 (20.3)		7.83 (5.75–10.66)	<0.001	10.28 (6.25–16.92)	<0.001
Years of education	0–9 years	86 (8.5)	131 (13.1)	109 (10.9)	98 (9.8)	58 (5.8)	<0.001	Ref.		Ref.	
	10–12 years	296 (29.5)	280 (28.0)	287 (28.7)	312 (31.2)	287 (28.7)		0.45 (0.33–0.61)	<0.001	0.96 (0.53–1.73)	0.878
	More than 12	623 (62.0)	590 (58.9)	605 (60.5)	590 (59.0)	654 (65.4)		0.60 (0.45–0.81)	<0.001	0.77 (0.44–1.36)	0.368
Health professionals		153 (15.2)	156 (15.6)	170 (17.0)	180 (18.0)	143 (14.3)	0.169	0.86 (0.69–1.08)	0.195	1.21 (0.84–1.75)	0.309
Area of residence	Rural	245 (24.4)	244 (24.4)	244 (24.4)	244 (24.4)	244 (24.4)	0.999	Ref.		Ref.	
	Urban	760 (75.6)	757 (75.6)	756 (75.6)	756 (75.6)	756 (75.6)		1.25 (1.04–1.51)	0.020	1.01 (0.75–1.36)	0.960

OR: Odds Ratio; CI: Confidence Interval.

**Table 2:** Preventive practices related to COVID-19 during each survey.

	Survey						Vaccination Intention		Vaccination or Intention to Be Vaccinated	
Categories	1	2	3	4	5	Comparison between Surveys	Surveys 1–4		Survey 5	
							OR 95%CI	Sig.	OR 95%CI	Sig.
Q14 (symptoms) (>5 correct answers)	888 (88.4)	892 (89.1)	875 (87.5)	907 (90.7)	882 (88.2)	0.201	2.24 (1.74–2.88)	<0.001	1.75 (1.19–2.56)	0.004
Q25. National recommendations to prevent COVID-19 infection are followed	683 (68.0)	594 (59.3)	658 (65.9)	628 (62.9)	634 (63.4)	0.001	18.52 (11.97–28.64)	<0.001	14.42 (5.86–35.48)	<0.001
Frequently washed my hands with soap and water for at least 20 s	931 (93.4)	908 (91.6)	926 (93.2)	927 (93.7)	914 (92.3)	0.358	3.01 (2.19–4.14)	<0.001	2.78 (1.57–4.91)	<0.001
Avoided touching my eyes, nose and mouth with unwashed hands	903 (91.1)	903 (91.5)	894 (90.4)	897 (90.3)	907 (91.7)	0.755	2.72 (2.08–3.54)	<0.001	4.63 (2.35–9.11)	<0.001
Used disinfectants to clean hands when soap and water were not available	958 (96.2)	955 (96.4)	958 (96.1)	953 (96.1)	947 (95.7)	0.949	4.41 (2.90–6.71)	<0.001	3.33 (1.60–6.96)	0.001
Remained at home when I was sick or had a cold	872 (94.8)	844 (93.0)	868 (94.1)	871 (95.1)	814 (90.5)	<0.001	2.63 (1.82–3.79)	<0.001	2.68 (1.42–5.06)	0.002
Used herbal supplements to prevent or treat COVID-19	335 (35.1)	336 (36.5)	289 (30.8)	313 (33.5)	282 (30.9)	0.030	0.38 (0.26–0.55)	<0.001	0.37 (0.28–0.49)	<0.001
Covered my mouth and/or nose when coughing or sneezing	977 (97.6)	971 (97.6)	976 (98.1)	976 (98.1)	951 (95.9)	0.009	2.78 (1.50–5.17)	<0.001	2.40 (1.17–4.91)	0.014
Being cautious when opening mail parcels and letters	503 (55.2)	497 (54.7)	457 (50.8)	530 (57.5)	462 (52.1)	0.038	2.30 (1.92–2.76	<0.001	1.60 (1.22–2.11)	0.001
Vaccinated for influenza	434 (45.5)	343 (36.1)	393 (42.0)	454 (47.9)	357 (38.3)	<0.001	7.80 (6.25–9.72)	<0.001	2.26 (1.72–2.98)	<0.001
Wore a face mask	975 (97.7)	957 (96.7)	971 (97.5)	959 (97.1)	962 (96.9)	0.623	7.21 (4.12–12.62)	<0.001	4.04 (2.13–7.66)	<0.001
Used antibiotics to prevent or treat COVID-19	129 (13.9)	121 (13.2)	110 (12.0)	115 (12.5)	122 (14.0)	0.672	0.55 (0.42–0.73)	<0.001	0.45 (0.33–0.63)	<0.001
Used homeopathic remedies to prevent or treat COVID-19	84 (9.2)	89 (10.1)	67 (7.5)	88 (9.9)	92 (10.7)	0.189	0.51 (0.37–0.71)	<0.001	0.46 (0.32–0.67)	<0.001
Ensured physical distancing in public areas	920 (92.6)	901 (90.8)	892 (90.9)	914 (92.6)	890 (90.8)	0.307	5.02 (3.73–6.75)	<0.001	19.74 (7.81–49.86)	<0.001
Self-isolation	695 (71.8)	648 (68.3)	717 (74.5)	689 (71.8)	627 (65.9)	<0.001	2.55 (2.13–3.06)	<0.001	2.28 (1.61–3.21)	<0.001
Disinfected surfaces	876 (87.7)	868 (88.0)	845 (85.3)	847 (86.3)	872 (88.3)	0.221	1.95 (1.54–2.46)	<0.001	2.85 (1.76–4.61)	<0.001
Consumed garlic, ginger and lemon	311 (33.8)	268 (30.6)	264 (29.8)	261 (28.7)	225 (25.9)	0.008	0.53 (0.44–0.64)	<0.001	0.59 (0.44–0.80)	0.001

OR: Odds Ratio; CI: Confidence Interval.

**Table 3:** Knowledge regarding the pandemic across the surveys.

	Survey						Vaccination Intention		Vaccination or Intention to Be Vaccinated	
Categories The Kind of Information I Need the Most Relates to…	1	2	3	4	5	Comparison between Surveys	Surveys 1–4		Survey 5	
							OR 95%CI	Sig.	OR 95%CI	Sig.
how to protect myself and my family from COVID-19	885 (88.1)	855 (85.4)	884 (88.4)	891 (89.1)	870 (87.0)	0.108	4.48 (3.51–5.71)	<0.001	2.43 (1.67–3.54)	<0.001
the symptoms associated with COVID-19.	848 (84.4)	803 (80.2)	814 (81.4)	832 (83.2)	823 (82.3)	0.132	4.28 (3.47–5.29)	<0.001	2.19 (1.56–3.04)	<0.001
people’s stories about how they deal with the pandemic situation	590 (58.7)	581 (58.0)	592 (59.2)	621 (62.1)	594 (59.4)	0.407	1.22 (1.03–1.44)	0.023	0.71 (0.55–0.92)	0.010
about emerging evidence and scientific advancements in the development of vaccines or treatments for COVID-19.	880 (87.6)	863 (86.2)	892 (89.2)	880 (88.0)	888 (88.8)	0.272	9.81 (7.50–12.84)	<0.001	4.88 (3.16–7.56)	<0.001
how to care for a person belonging to a vulnerable group	843 (83.9)	817 (81.6)	824 (82.4)	866 (86.6)	804 (80.4)	0.003	2.24 (1.81–2.77)	<0.001	2.08 (1.52–2.85)	<0.001
how I can protect and ensure continuity of my children’s education	575 (57.2)	598 (59.7)	556 (55.6)	595 (59.5)	615 (61.5)	0.066	0.90 (0.76–1.06)	0.212	1.01 (0.78–1.31)	0.934
The difference between COVID-19 and influenza	820 (81.6)	770 (76.9)	773 (77.3)	795 (79.5)	782 (78.2)	0.072	3.21 (2.64–3.90)	<0.001	2.89 (2.13–3.94)	<0.001
about the evolution of the pandemic situation globally	886 (88.2)	843 (84.2)	864 (86.4)	853 (85.3)	848 (84.8)	0.093	4.91 (3.89–6.20)	<0.001	3.58 (2.49–5.14)	<0.001
about the evolution of the pandemic situation in Greece	886 (88.2)	856 (85.5)	899 (89.9)	870 (87.0)	878 (87.8)	0.049	6.85 (5.29–8.87)	<0.001	3.44 (2.32–5.11)	<0.001
informed about competent authorities’ decisions related to COVID19 public health measures	892 (88.8)	855 (85.4)	872 (87.2)	860 (86.0)	831 (83.1)	0.005	4.08 (3.20–5.19)	<0.001	2.43 (1.74–3.40)	<0.001
how the pandemic can affect my financial situation	854 (85.0)	865 (86.4)	869 (86.9)	869 (86.9)	835 (83.5)	0.128	1.25 (0.98–1.58)	0.069	1.13 (0.80–1.59)	0.487
how to maintain my mental health and well-being during isolation	750 (74.6)	773 (77.2)	746 (74.6)	769 (76.9)	751 (75.1)	0.474	1.58 (1.31–1.90)	<0.001	1.44 (1.08–1.93)	0.013
how to maintain my social contacts despite practicing physical distancing	673 (67.0)	660 (65.9)	695 (69.5)	710 (71.0)	687 (68.7)	0.112	1.33 (1.12–1.59)	0.001	1.43 (1.09–1.88)	0.009

OR: Odds Ratio; CI: Confidence Interval.

**Table 4:** Multivariate analysis, vaccination intention, data from surveys 1–4.

Variables	Vaccination Intention		
	Agree/Disagree		
	Sig.	aOR	95% CI
Age (years)	0.016	1.011	1.002–1.019
Gender (male/female)	<0.001	2.07	1.60–2.69
Education (10–12/0–9 years)	0.017	0.56	0.34-0.90
Education (>12/0–9 years)	0.627	0.89	0.56–1.42
Chronic illness	0.186	1.24	0.90–1.72
Area of residence (>500,000/<500,000)	0.145	1.22	0.94–1.58
Knowledge of symptoms (>5/≤5)	0.008	1.69	1.15–2.50
Knowledge about prevention	0.935	0.99	0.74–1.31
Influenza vaccination	<0.001	4.84	3.61–6.50
I follow the recommendations of my country’s authorities to prevent the spread of the new coronavirus (Agree/Disagree).	<0.001	3.97	1.90–8.29
I follow the recommendations of my country’s authorities to prevent the spread of the new coronavirus (NAND/Disagree)	0.001	1.63	1.24–2.14
Mass media and Social media	<0.001	1.43	1.25–1.62
HCWs and states’ instructions	<0.001	3.40	2.92–3.96
Family’s, friends’ and colleagues’ opinion	0.015	0.86	0.76–0.97
My and my loved ones’ wellbeing and health system overload	<0.001	1.62	1.42–1.86
Country’s economic recession	0.013	1.19	1.04–1.36
Personal financial problems	0.001	0.79	0.69–0.90
Losing my social life	0.048	0.88	0.78–0.99
Survey 4	Ref.		
Survey 1	0.004	0.60	0.42–0.85
Survey 2	0.345	0.85	0.60–1.20
Survey 3	0.628	1.09	0.77–1.55

NAND: Neither agree nor disagree; aOR: adjusted Odds Ratio; CI: Confidence Interval.

**Table 5:** Multivariate analysis, vaccination intention, data from survey 5.

Variables	Vaccination or Intention to Be Vaccinated		
	Yes/No		
	Sig.	aOR	95% CI
Age (years)	<0.001	1.04	1.03–1.05
Gender (male/female)	0.008	1.66	1.14–2.40
Education (10–12/0–9 years)	0.189	1.74	0.76–3.96
Education (>12/0–9 years)	0.565	1.26	0.57–2.80
Chronic illness	0.582	0.88	0.57–1.37
Area of residence (>500,000/<500,000)	0.040	1.49	1.02–2.17
Knowledge of symptoms (>5/≤5)	0.542	0.84	0.49–1.46
Influenza vaccination	<0.001	3.49	2.34–5.20
I follow the recommendations of my country’s authorities to prevent the spread of the new coronavirus (Agree/Disagree)	0.008	10.28	3.19–33.12
I follow the recommendations of my country’s authorities to prevent the spread of the new coronavirus (NAND/Disagree)	<0.001	4.88	1.51–15.75
HCWs, states’ instructions and public state mass media	<0.001	2.23	1.79–2.79
Private owned Mass media and Social media	0.599	1.05	0.88–1.26
Family’s, friends’ and colleagues’ opinion	0.048	0.83	0.68–1.00
My and my loved ones’ wellbeing	0.061	1.21	0.99–1.47
Country’s economic recession and health system overload	0.048	1.21	1.00–1.47
Losing my social life	0.688	0.96	0.79–1.17
Personal financial problems	0.010	0.77	0.63–0.94

aOR: adjusted Odds Ratio; CI: Confidence Interval.

## Data Availability

The datasets used and/or analyzed during the current study are available from the National Ministry of Health in collaboration with the National Ministry for climate crisis and civil protection.

## Supplementary Materials

The following supporting information can be downloaded at: https://www.mdpi.com/article/10.3390/ijerph20010216/s1.
