# Supplementary material for: Behavioral and Cultural Insights, a Nationwide Study Based on Repetitive Surveys of WHO Behavioral Insights Tool in Greece Regarding COVID-19 Pandemic and Vaccine Acceptance
Source: Int J Environ Res Public Health. 2022 Dec 23;20(1):216. doi: 10.3390/ijerph20010216 (PMC9819918; doi:10.3390/ijerph20010216)
Supplement: Supplementary file 1 [file ijerph-20-00216-s001.zip › ijerph-1888246-supplementary.pdf]

Supplementary material

List of variables

ID

Q1. How old are you? I am ….

Q2. What is your gender?

1. Male
2. Female
3. Other

Q3. How many years of education have you completed?

1. 0-9 years of education.
2. 9-12 years of education (Junior High or High school graduate).
3. >=12 years of education.

Q4. Are you a health professional?

1. No
2. Nurse
3. Pharmacist
4. Doctor
5. Other

Q5. Do you have a chronic illness?

1. Yes
2. No
3. I don't Know

Q6. How many people live in the village or city where you live?

1. < 5,000 residents.
2. 5,001 - 20,000 residents.
3. 20,001 - 100,000 residents.
4. 100,001 - 500,000 residents.
5. > 500,000 residents.

Q7. Where do you live?

1. Rural area.

2. Urban area.

Q8. To your knowledge, are you, or have you been infected with COVID-19?

1. No, I got tested and the results were negative.
2. No
3. Yes, I got tested and the results were positive.
4. Yes, I was suspicious but I did not confirm it with a test.
5. I don't know.

Q9. Do you know people from your close social circle who are or have been infected with COVID-19 (suspected or confirmed)?

1. Yes, conirmed by test resuls.
2. Yes, suspicious but never confirmed by test results.
3. No, tested but the results were negative.
4. No
5. Don't know.

Q10. Which of these below could be symptoms of COVID-19?

1. Fever.
2. Cough.
3. Difficulty in breathing.
4. Sore throat.
5. A runny or stuffy nose.
6. Myalgia or body aches.
7. Headaches.
8. Fatigue.
9. Diarrhea.
10. Loss of taste or smell.

Q11. How severe would contracting COVID-19 be for you (how seriously ill do you think you will be)? 1 - not severe … 7 - Very severe.

Next, we would like to know about you own practices related to COVID-19.

Q12. Which of the following measures have you taken to prevent infection from COVID-19?
Please choose all the meausres that apply to you regardless if you have already taken them or not.

1. Frequently washed my hands with soap and water for at least 20 seconds.
2. Avoided touching my eyes, nose and mouth with unwashed hands.
3. Used disinfectants to clean hands when soap and water were not available.

4. Stayed at home while being sick or having the flu.
5. Took natural supplements.
6. Covered my mouth or nose while sneezing or couhing.
7. Been cautious when opening letters.
8. Got flu vaccinated.
9. Used a face mask.
10. Used antibiotics.
11. Used homeopathic treatments.
12. Kept social distances (of at least 2m between me and someone else while being outside).
13. Self-isolated.
14. Surface disinfection.
15. Cell phone disinfection.
16. Ate garlic, ginger or lemons.
17. Other precautionary measure, please specify...

Q13. I'm following my country's guidelines to prevent COVID-19 from spreading further. 1 - Not at all … 7 - A Lot

Q14. How much do you trust information about COVID-19 from the following sources?

1. State television chanels.
2. Everyday or weekly newspapers.
3. Conversations with family and friends.
4. Conversations with co-workers.
5. Consultations with health workers.
6. Privately owned television channels.
7. News websites.
8. Social media.
9. Privately owned radio stations.
10. State radio stations.
11. Government's formal news reports.
12. News reports from health institutions.
13. Polls.
14. Celebrities and infuencers.

Q15. Are there any other sources of information you may trust for updates on COVID-19?

1. If yes, please note:
2. No, there is no other source I trust.

Q16. How much trust do you have towards this source of informaion (if you checked yes in question 15)?

1 - I trust it a little … 7 - I trust it a lot.

Q17. The kind of information I need the most relates to…

1. The symptoms of Covid-19.
2. How can I protect myself and my famly from COVID-19.
3. Other's personal stories on how they are dealing with the pandemic.
4. Scientific progress on developing a vaccine or any other kind of treatment against COVID-19.
5. How to take care of someone who belongs to a high risk group.
6. How to take care of my child's or children's education in the best possible way.
7. What's the difference between COVID-19 and other illnesses like the flu?
8. The pandemic's evolution around he world.
9. The pandemic's evolution in Greece.
10. Information regarding authorities' decisions.
11. How will the pandemic affect me financially?
12. How to maintain my mental health during isolation.
13. How to retain my social contacts despite being apart.
14. Other.

Please state other (from the previous question) regarding the kid of information you need the most.

Q18. How frequently do you read the news regarding COVID-19?

1 - Never … 7 - Many times per day.

Q19. Please state your opinion regarding the statements bellow:

1. If a vaccine becomes available and they suggest it, I'll do it.

Q20. Opinions often include fears and worries. Right now how much do you worry about…

1. The loss of a beloved person?
2. The already encumbered health system?
3. Your own mental health?
4. Your own physical health?
5. The health of the ones you love?

6. The restrictions on travelling and transportation?
7. Lost vacation chances.
8. Small companies being shut down?
9. The economic downturn of your own country?
10. Restricted access to food?
11. The possibility of being unemployed?
12. Being unable to pay bills on time?
13. Being unable to visit someone who depends on you?
14. Being able to defend your decision to not attend a social gathering that your family or friends will attend?

Q21. Is there anything else that worries you at the moment?

a. If yes, please state:

b. No, there's nothing else.

Wave 5

Q22. Have you been vaccinated against COVID-19? 1 - Yes … 2 - NO

Table S1. Socio-demographic characteristics and vaccination intention (NAND/Disagree)

		Vaccination intention	
		Surveys 1-4	
Variable	Categories	NAND/Disagree OR 95%CI	Sig.
Gender	Male	0.85 (0.72-1.02)	0.077
	Female	Ref.	
Age groups	18-30	Ref.	
	31-50	0.88 (0.71-1.10)	0.138
	51-65	0.85 (0.66-1.09)	0.189
	66-90	0.90 (0.64-1.26)	0.536
Years of education	0-9 years	Ref.	
	10-12 years	1.03 (0.72-1.46)	0.879
	More than 12	1.22 (0.88-1.71)	0.235
Health professionals		1.16 (0.92-1.46)	0.208
Area of residence	Rural	Ref.	
	Urban	1.38 (1.14-1.68)	0.001

NAND: Neither agree nor disagree; OR: Odds Ratio; CI: Confidence Interval

Table S2. Infection and vaccination intention in each survey.

Categories	Survey 1 N (%)	Survey 2 N (%)	Survey 3 N (%)	Survey 4 N (%)	Survey 5 N (%)	Comparison between surveys	Association with COVID-19 vaccination intention (Surveys 1-4)				Vaccination intention (Survey 5)	
							Agree/Disagree		NAND/Disagree			
							OR 95%CI	Sig.	OR 95%CI	Sig.	OR 95%CI	Sig.
Q11. To your knowledge, are you, or have you been, infected with COVID-19? (Yes, confirmed by test)	29 (3.0)	33 (3.5)	35 (3.6)	53 (5.5)	54 (5.4)	0.006	1.12 (0.69-1.81)	0.656	1.63 (1.00-2.65)	0.047	0.41 (0.23-0.75)	0.003
Q12. Do you know people in your immediate social environment who are or have been infected with COVID-19? (Yes, confirmed by test)	442 (44.0)	458 (45.8)	517 (51.7)	590 (59.0)	580 (58.0)	<0.001	1.29 (1.09-1.53)	0.003	0.92 (0.77-1.10)	0.347	0.98 (0.76-1.26)	0.878

NAND: Neither agree nor disagree; OR: Odds Ratio; CI: Confidence Interval

Table S3. Concern of a severe illness among the surveys

	Surveys	Q42_1. If a COVID-19 vaccine becomes available and it is recommended to me, I will get vaccinated.	Mean	Std. Deviation	Sig
Q19. How severe would contracting COVID-19 be for you (how seriously ill do you think you will be)?<br>Not severe 1 2 3 4 5 6 7 Very severe	Survey 1	No	3.42	1.932	<0.001
		Yes	4.65	1.782	
	Survey 2	No	3.31	1.878	<0.001
		Yes	4.33	1.693	
	Survey 3	No	3.15	1.718	<0.001
		Yes	4.71	1.674	
	Survey 4	No	2.93	1.752	<0.001
		Yes	4.33	1.783	
	Survey 5	No	3.52	1.691	<0.001
		Yes	4.32	1.766	

Table S4. Preventive practices related to COVID-19 and vaccination intention (NAND/Disagree)

Categories	Vaccination intention (Surveys 1-4) NAND/Disagree	
	OR 95%CI	Sig.
Q14 (symptoms) (>5 correct answers)	1.18 (1.02-1.36)	0.031
Q25. National recommendations to prevent COVID-19 infection are followed	7.71 (5.10-11.66)	<0.001
Frequently washed my hands with soap and water for at least 20 seconds	1.38 (0.54-0.97)	0.032
Avoided touching my eyes, nose and mouth with unwashed hands	2.00 (1.53-2.62)	<0.001
Used disinfectants to clean hands when soap and water were not available	2.06 (1.42-2.99)	<0.001
Remained at home when I was sick or had a cold	1.21 (0.86-1.70)	0.265
Used herbal supplements to prevent or treat COVID-19	0.92 (0.77-1.11)	0.396
Covered my mouth and/or nose when coughing or sneezing	0.81 (0.48-1.35)	0.414
Being cautious when opening mail parcels and letters	1.95 (1.62-2.36)	<0.001
Vaccinated for influenza	2.90 (2.30-3.66)	<0.001
Wore a face mask	1.95 (1.28-2.67)	0.001
Used antibiotics to prevent or treat COVID-19	1.58 (1.22-2.04)	<0.001
Used homeopathic remedies to prevent or treat COVID-19	1.27 (0.95-1.71)	0.107
Ensured physical distancing in public areas	2.22 (1.70-2.90)	<0.001
Self-isolation	2.13 (1.76-2.57)	<0.001
Disinfected surfaces	1.81 (1.42-2.30)	<0.001
Consumed garlic, ginger and lemon	0.75 (0.62-0.91)	0.004

NAND: Neither agree nor disagree; OR: Odds Ratio; CI: Confidence Interval

Table S5. Knowledge regarding the pandemic and vaccination intention (NAND/Disagree)

Categories<br>The kind of information I need the most relates to…	Vaccination intention (Surveys 1-4)<br>NAND/Disagree<br>OR 95%CI	Sig.
how to protect myself and my family from COVID-19	2.19 (1.74-2.74)	<0.001
the symptoms associated with COVID-19.	2.05 (1.68-2.51)	<0.001
people's stories about how they deal with the pandemic situation	1.18 (0.98-1.40)	0.075
about emerging evidence and scientific advancements in the development of vaccines or treatments for COVID-19.	1.88 (1.69-2.09)	<0.001
how to care for a person belonging to a vulnerable group	1.55 (1.25-1.92)	<0.001
how I can protect and ensure continuity of my children's education	1.16 (0.97-1.38)	0.112
The difference between COVID-19 and influenza	1.82 (1.50-2.21)	<0.001
about the evolution of the pandemic situation globally	2.21 (1.78-2.75)	<0.001
about the evolution of the pandemic situation in Greece	2.33 (1.87-2.91)	<0.001
informed about competent authorities' decisions related to COVID19 public health measures	1.70 (1.36-2.12)	<0.001
how the pandemic can affect my financial situation	1.08 (0.84-1.38)	0.562
how to maintain my mental health and well-being during isolation	1.60 (1.32-1.96)	<0.001
how to maintain my social contacts despite practicing physical distancing	1.28 (1.06-1.54)	0.009

NAND: Neither agree nor disagree; OR: Odds Ratio; CI: Confidence Interval

Table S6. Sources of information, comparison between age groups (“less than 40” vs “40 and above”)

Categories	Survey 1 N (%)	Survey 2 N (%)	Survey 3 N (%)	Survey 4 N (%)	Survey 5 N (%)	Comparison between surveys	Association with COVID-19 vaccination intention (Agree/Disagree) (Surveys 1-4)				Vaccination intention (Survey 5)			
							**(≤40)** OR 95%CI	Sig.	**(>40)** OR 95%CI	Sig.	**(≤40)** OR 95%CI	Sig.	**(>40)** OR 95%CI	Sig.
Q40. How often do you seek information about COVID-19?(Several times a day)	418 (41.6)	356 (35.6)	387 (38.7)	378 (37.8)	312 (31.2)	<0.001	15.85 (8.75-28.70)	<0.001	11.59 (7.58-17.74)	<0.001	3.16 (1.33-7.52)	0.008	4.28 (2.16-8.52)	<0.001
Q37_1. State owned television network	167 (31.5)	98 (18.7)	133 (24.8)	109 (20.5)	114 (11.4)	<0.001	5.53 (2.69-11.35)	<0.001	20.29 (12.32-33.41)	<0.001	0.78 (0.23-2.62)	0.682	13.65 (5.73-32.49)	<0.001
Q37_2. Daily or weekly newspapers (printed)	114 (25.3)	66 (14.9)	67 (15.2)	54 (11.5)	58 (5.8)	<0.001	4.75 (2.40-9.41)	<0.001	16.90 (8.19-34.88)	<0.001	2.46 (0.66-9.09)	0.167	24.49 (3.32-180.8)	<0.001
Q37_3. Opinions of family and friends	164 (48.5)	129 (37.9)	181 (48.8)	184 (46.9)	151 (15.1)	0.005	0.51 (0.33-0.80)	0.003	1.43 (1.02-2.02)	0.040	0.87 (0.41-1.84)	0.706	1.22 (0.67-2.23)	0.139
Q37_4. Opinions of Colleagues	89 (25.7)	67 (19.5)	87 (24.9)	84 (22.0)	80 (8.0)	0.406	1.32 (0.77-2.25)	0.315	1.16 (0.80-1.68)	0.446	1.96 (0.85-4.51)	0.111	1.85 (0.85-4.05)	0.120
Q37_5. Health care workers	465 (81.3)	382 (76.4)	441 (84.8)	462 (82.6)	411 (41.1)	0.001	19.06 (11.26-32.27)	<0.001	18.56 (12.68-27.17)	<0.001	4.78 (2.16-10.60)	<0.001	12.53 (6.84-22.98)	<0.001

Q37_6. Private owned television network	100 (19.3)	51 (9.6)	84 (15.9)	60 (10.9)	77 (7.7)	<0.001	8.78 (3.02-25.51)	<0.001	10.42 (5.94-18.28)	<0.001	1.90 (0.56-6.43)	0.298	5.99 (2.49-14.38)	<0.001
Q37_7. Online newspapers and websites	73 (16.6)	62 (14.7)	66 (15.1)	67 (13.8)	58 (5.8)	0.080	2.29 (1.30-4.06)	0.004	1.58 (1.02-2.45)	<0.001	0.75 (0.22-2.57)	0.643	1.20 (0.58-2.51)	0.623
Q37_8. Social media	48 (9.1)	39 (7.4)	62 (11.6)	55 (9.7)	43 (4.3)	0.046	1.63 (0.86-3.11)	0.135	1.06 (0.66-1.68)	0.817	0.52 (0.13-2.01)	0.512	0.99 (0.43-2.29)	0.986
Q37_9. Privately owned radio network	74 (16.7)	48 (10.5)	66 (14.1)	35 (7.3)	49 (4.9)	0.001	8.73 (3.00-25.37)	<0.001	4.47 (2.64-7.58)	<0.001	-	<0.001	4.08 (1.55-10.75)	0.002
Q37_10. State owned radio network	123 (26.4)	69 (15.3)	89 (20.1)	93 (19.1)	93 (9.3)	0.003	7.77 (3.36-17.98)	<0.001	12.48 (7.53-20.66)	<0.001	3.56 (1.02-12.36)	0.035	10.28 (4.01-26.40)	<0.001
Q37_11. Ministry of Health	285 (51.7)	191 (36.9)	240 (42.5)	228 (39.7)	232 (23.2)	<0.001	12.00 (7.24-19.88)	<0.001	24.43 (16.50-36.17)	<0.001	3.20 (1.58-6.46)	0.001	21.55 (10.32-45.03)	<0.001
Q37_12. Health care institutions	393 (74.4)	311 (63.6)	345 (69.7)	351 (67.6)	357 (35.7)	0.017	21.80 (13.41-35.45)	<0.001	21.98 (15.33-31.52)	<0.001	4.20 (2.12-8.32)	<0.001	23.45 (12.45-44.18)	<0.001
Q37_13. Opinion poll	132 (27.0)	85 (18.7)	105 (22.2)	93 (19.1)	88 (8.8)	0.003	4.27 (2.32-7.85)	<0.001	7.42 (4.78-11.53)	<0.001	1.64 (0.62-4.30)	0.313	5.61 (2.55-12.34)	<0.001
Q37_14. VIPs	44 (6.9)	35 (5.5)	37 (5.8)	20 (2.9)	22 (2.2)	0.001	6.13 (1.37-27.41)	0.007	3.41 (1.69-6.89)	<0.001	1.39 (0.09-22.53)	0.815	3.18 (0.72-14.07)	0.108

NAND: Neither agree nor disagree; OR: Odds Ratio; CI: Confidence Interval

Table S7. Factor analysis in sources of information, surveys 1-4

Surveys 1-4		Component			
Factor	Trust …	1	2	3	Eigenvalues
Mass media and social media	Privately-owned radio	0.708	0.404		6.599
	VIPs	0.692			
	Social media	0.677		0.361	
	Privately-owned television	0.676	0.505		
	General interest Websites	0.671			
	State-owned radio	0.649	0.548		
	Printed newspapers	0.645	0.474		
	State-owned televison	0.616	0.613		
	Opinion polls	0.528	0.508		
HCWs and states' instructions	Healthcare institutions		0.829		1.685
	Healthcare workers		0.809		
	Ministry of Health	0.392	0.769		
Family's, friends' and colleagues' opinion	Familly and friends			0.832	1.07
	Colleagues			0.808	
Extraction Method: Principal Component Analysis. Rotation Method: Varimax with Kaiser Normalization.					
a. Rotation converged in 10 iterations.					

Table S8. Factor analysis regarding concerns during lockdown period, surveys 1-4

Surveys 1-4		Component				Eigenvalues
Factor	I am worried about…	1	2	3	4	
My and my loved ones' wellbeing and health system overload	losing a loved one	0.785				4.55
	the health of my loved ones	0.777	0.322			
	my physical health	0.749				
	health system overload	0.599	0.505			
	my mental health	0.481		0.409	0.357	
	the inability to visit people dependent on me for care	0.460			0.400	
Country's economic recession	the economic recession		0.823			1.984
	small businesses closures		0.807			
Personal financial problems	becoming unemployed			0.860		1.285
	the inability to cover household expenses			0.840		
	limited access to food			0.449	0.386	
Losing my social life	missing vacations				0.794	1.071
	having to defend my decision not to participate in a social event that my family or friends expect me to attend				0.664	
	going outside		0.443		0.607	
Extraction Method: Principal Component Analysis. Rotation Method: Varimax with Kaiser Normalization.						
a. Rotation converged in 8 iterations.						

Table S9. Factor analysis in sources of information, 5th survey

5th Survey		Component			Eigenvalues
Factor	Trust …	1	2	3	
HCWs, states' instructions and public state mass media	Healthcare institutions	0.841			6.423
	Ministry of Health	0.807			
	Healthcare workers	0.774			
	State-owned televison	0.691	0.520		
	State-owned radio	0.611	0.587		
	Opinion polls	0.598	0.407		
Private owned mass media and social media	Social media		0.755		1.746
	Websites		0.700		
	Privately-owned radio	0.413	0.683		
	VIPs		0.657		
	Printed newspapers	0.497	0.648		
	Privately-owned television	0.534	0.633		
Family's, friends' and colleagues' opinion	Familly and friends			0.835	1.179
	Colleagues			0.827	
Extraction Method: Principal Component Analysis. Rotation Method: Varimax with Kaiser Normalization.					
a. Rotation converged in 7 iterations.					

Table S10. Factor analysis regarding concerns during lockdown period, 5th survey

5th Survey		Component				
Factor	I am worried about…	1	2	3	4	Eigenvalues
My and my loved ones' wellbeing	my physical health	0.763				4.603
	the health of my loved ones	0.754	0.382			
	losing a loved one	0.743	0.341			
	the inability to visit people dependent on me for care	0.555		0.315		
	my mental health	0.554		0.347	0.356	
Country's economic recession and health system overload	small businesses closures		0.825			1.929
	the economic recession		0.820			
	health system overload	0.443	0.628			
Losing my social life	missing vacations			0.794		1.341
	going outside		0.359	0.664		
	having to defend my decision not to participate in a social event that my family or friends expect me to attend	0.337		0.608		
	limited access to food	0.302		0.516	0.385	
Personal financial problems	becoming unemployed				0.854	1.011
	the inability to cover household expenses				0.796	
Extraction Method: Principal Component Analysis.<br>Rotation Method: Varimax with Kaiser Normalization.						
a. Rotation converged in 10 iterations.						

Table S11. Factors of sources of information in surveys 1 to 4

Vaccination intention	Sources of information	<=40 years old			>40 years old		
		Sig.	OR	95%CI	Sig.	OR	95%CI
Agree/ Disagree	Mass media and Social media	<0.001	1.49	1.27-1.74	<0.001	1.62	1.41-1.86
	HCWs and states' instructions	<0.001	6.06	4.90-7.49	<0.001	5.69	4.84-6.68
	Family's, friends' and colleagues' opinion	0.084	0.87	0.74-1.02	0.053	0.88	0.78-1.00
NAND/ Disagree	Mass media and Social media	<0.001	1.85	1.60-2.14	<0.001	1.67	1.44-1.93
	HCWs and states' instructions	<0.001	2.66	2.23-2.23	<0.001	2.32	2.00-2.70
	Family's, friends' and colleagues' opinion	0.052	0.87	0.76-1.00	<0.001	0.75	0.66-0.85
NAND: Neither agree nor disagree; OR: Odds Ratio; CI: Confidence Interval							

Table S12. Factors of sources of information in survey 5

Vaccination	Sources of information	<=40 years old			>40 years old		
		Sig.	OR	95%CI	Sig.	OR	95%CI
Yes/No	HCWs, states' instructions and public state mass media	<0.001	2.34	1.75-3.12	<0.001	3.40	2.71-4.28
	Private owned Mass media and social media	0.383	0.91	0.73-1.13	0.855	0.98	0.79-1.21
	Family's, friends' and colleagues' opinion	0.284	1.13	0.90-1.41	0.293	0.89	0.72-1.10
OR: Odds Ratio; CI: Confidence Interval							

Table S13. Psychosocial variables in each survey.

Categories<br>I am worried about…		Survey 1<br>N (%)	Survey 2<br>N (%)	Survey 3<br>N (%)	Survey 4<br>N (%)	Survey 5<br>N (%)	Comparison between surveys
Q48_1. …losing a loved one	Agree	522 (51.9)	481 (48.1)	555 (55.5)	530 (53.0)	484 (48.4)	0.001
	NAND	378 (37.6)	417 (41.7)	338 (33.8)	340 (34.0)	390 (39.0)	
	Disagree	105 (10.4)	103 (10.3)	107 (10.7)	130 (13.0)	126 (12.6)	
Q48_2. … health system overload	Agree	553 (55.0)	526 (52.5)	670 (67.0)	630 (63.0)	547 (54.7)	<0.001

	NAND	395 (39.3)	404 (40.4)	283 (28.3)	306 (30.6)	397 (39.7)	
	Disagree	57 (5.7)	71 (7.1)	47 (4.7)	64 (6.4)	56 (5.6)	
Q48_3. … my mental health	Agree	253 (25.3)	299 (29.9)	330 (33.0)	270 (27.0)	281 (28.1)	0.002
	NAND	469 (46.7)	455 (45.5)	408 (40.8)	444 (44.4)	430 (43.0)	
	Disagree	282 (28.1)	246 (24.6)	262 (26.2)	286 (28.6)	289 (28.9)	
Q48_4. … my physical health	Agree	344 (34.2)	300 (30.0)	347 (34.7)	344 (34.4)	296 (29.6)	0.114
	NAND	495 (49.3)	534 (53.3)	471 (47.1)	480 (48.0)	507 (50.7)	
	Disagree	166 (16.5)	167 (16.7)	182 (18.2)	176 (17.6)	197 (19.7)	
Q48_5. …the health of my loved ones	Agree	570 (56.7)	533 (53.2)	622 (62.2)	617 (61.7)	541 (54.0)	<0.001
	NAND	378 (37.6)	392 (39.2)	317 (31.7)	319 (31.9)	380 (38.0)	
	Disagree	57 (5.7)	76 (7.6)	62 (6.2)	64 (6.2)	80 (8.0)	
Q48_6. …going outside	Agree	421 (41.9)	441 (44.1)	472 (47.2)	415 (41.5)	337 (33.7)	0.088
	NAND	449 (44.7)	433 (43.3)	389 (38.9)	437 (43.7)	478 (47.8)	
	Disagree	135 (13.4)	127 (12.7)	139 (13.9)	148 (14.8)	185 (18.5)	
Q48_7. …missing vacations	Agree	227 (22.6)	286 (28.6)	285 (28.5)	270 (27.0)	244 (24.4)	0.013
	NAND	439 (43.7)	418 (41.8)	399 (39.9)	391 (39.1)	442 (44.2)	
	Disagree	339 (33.7)	297 (29.7)	316 (31.6)	339 (33.9)	314 (31.4)	
Q48_8. …small businesses closures	Agree	652 (64.9)	636 (63.5)	712 (71.2)	667 (66.7)	616 (61.6)	<0.001
	NAND	321 (31.9)	318 (31.7)	260 (26.0)	280 (28.0)	342 (34.2)	
	Disagree	32 (3.2)	48 (4.8)	28 (2.8)	53 (5.3)	42 (4.2)	
Q48_9.…the economic recession	Agree	669 (66.5)	669 (66.8)	739 (73.9)	667 (66.7)	638 (63.8)	<0.001
	NAND	302 (30.0)	293 (29.3)	236 (23.6)	280 (28.0)	327 (32.7)	

	Disagree	35 (3.5)	39 (3.9)	25 (2.5)	53 (5.3)	35 (3.5)	
Q48_10. …limited access to food	Agree	137 (13.6)	132 (13.2)	135 (13.5)	140 (14.0)	124 (12.4)	0.028
	NAND	401 (39.9)	412 (41.2)	336 (33.6)	364 (36.4)	387 (38.7)	
	Disagree	467 (46.5)	457 (45.7)	528 (52.9)	497 (49.7)	489 (48.9)	
Q48_11. …becoming unemployed	Agree	329 (32.7)	378 (37.8)	360 (36.0)	309 (30.9).	317 (31.7)	<0.001
	NAND	331 (32.9)	354 (35.4)	265 (26.5)	271 (27.1)	309 (30.9)	
	Disagree	345 (34.3)	269 (26.9)	375 (37.5)	420 (42.0)	374 (37.4)	
Q48_12. …the inability to cover household expenses	Agree	341 (34.0)	365 (36.5)	383 (38.3)	302 (30.2)	348 (34.8)	<0.001
	NAND	385 (38.3)	427 (42.7)	353 (35.3)	375 (37.5)	407 (40.7)	
	Disagree	278 (27.7)	209 (20.9)	264 (26.4)	323 (32.3)	246 (24.6)	
Q48_13. …the inability to visit people dependent on me for care	Agree	335 (33.3)	370 (37.0)	379 (37.9)	359 (35.9)	292 (29.2)	<0.001
	NAND	456 (45.4)	443 (44.3)	390 (39.0)	424 (42.4)	467 (46.7)	
	Disagree	214 (21.3)	188 (18.8)	232 (23.2)	218 (21.8)	241 (24.1)	
Q48_14. having to defend my decision not to participate in a social event that my family or friends expect me to attend	Agree	168 (16.7)	175 (17.5)	211 (21.1)	171 (17.1)	162 (16.2)	0.011
	NAND	425 (42.3)	456 (45.5)	375 (37.5)	424 (42.4)	435 (43.5)	
	Disagree	412 (41.0)	371 (37.0)	415 (41.5)	405 (40.5)	403 (40.3)	
NAND: Neither agree nor disagree							

Table S14. Factors of psychosocial variables

Vaccination or intention to be vaccinated	Variables	OR	95% CI	Sig.
Agree/ Disagree	My and my loved ones' wellbeing and health system overload	2.83	2.56-3.13	<0.001
	Country's economic recession	1.19	1.09-1.31	<0.001
	Personal financial problems	0.51	0.49-0.56	<0.001
	Losing my social life	0.71	0.64-0.78	<0.001
NAND/ Disagree	My and my loved ones' wellbeing and health system overload	1.62	1.48-1.78	<0.001
	Country's economic recession	0.72	0.66-0.79	<0.001
	Personal financial problems	0.89	0.80-0.99	0.027
	Losing my social life	1.02	0.92-1.12	0.739
NAND: Neither agree nor disagree; OR: Odds Ratio; CI: Confidence Interval				

Table S15. Factors of psychosocial variables

Vaccination	Variables	OR	95% CI	Sig.
Yes/No	My and my loved ones' wellbeing	1.63	1.42-1.88	<0.001
	Country's economic recession and health system overload	1.51	1.32-1.73	<0.001
	Personal financial problems	0.76	0.66-0.87	<0.001
	Losing my social life	0.58	0.50-0.67	<0.001
OR: Odds Ratio; CI: Confidence Interval				

Table S16. Multivariate analysis, neutral position on COVID-19 vaccination, data from surveys 1-4.

Variables	NAND/ Disagree		
	Sig.	aOR	95% C.I.
Age (years)	0.475	0.997	0.990-1.005
Gender (male/female)	0.328	1.12	0.90-1.40
Education (10-12/0-9 years)	0.826	1.05	0.67-1.64
Education (>12/0-9 years)	0.106	1.43	0.93-2.19
Chronic illness	0.781	1.04	0.78-1.39
Area of residence (>500,000/<500,000)	0.019	1.32	1.05-1.65
Knowledge of symptoms (>5/<=5)	0.054	1.37	1.00-1.88
Knowledge about prevention	0.787	0.97	0.76-1.23
Influenza vaccination	<0.001	2.61	1.98-3.43
I follow the recommendations of my country's authorities to prevent the spread of the new coronavirus (Agree/Disagree).	<0.001	2.92	1.69-5.04
I follow the recommendations of my country's authorities to prevent the spread of the new coronavirus (NAND/Disagree)	<0.001	2.76	1.64-4.66
Mass media and Social media	<0.001	1.63	1.44-1.84
HCWs and states' instructions	<0.001	2.21	1.92-2.56
Family's, friends' and colleagues' opinion	0.016	0.87	0.78-0.98
My and my loved ones' wellbeing and health system overload	0.001	1.23	1.09-1.39
Country's economic recession	<0.001	0.77	0.69-0.86
Personal financial problems	0.865	1.01	0.89-1.14

Losing my social life	0.201	1.08	0.96-1.21
Survey 4		Ref.	
Survey 1	0.170	1.24	0.91-1.69
Survey 2	0.058	1.35	0.99-1.83
Survey 3	0.002	1.66	1.21-2.29

NAND: Neither agree nor disagree; aOR: adjusted Odds Ratio; CI: Confidence Interval
